# Implementation and fidelity of a participatory learning and action cycle intervention to prevent and control type 2 diabetes in rural Bangladesh

**DOI:** 10.1186/s41256-019-0110-6

**Published:** 2019-07-05

**Authors:** Joanna Morrison, Kohenour Akter, Hannah Maria Jennings, Abdul Kuddus, Tasmin Nahar, Carina King, Sanjit Kumer Shaha, Naveed Ahmed, Hassan Haghparast-Bidgoli, Anthony Costello, A. K. Azad Khan, Kishwar Azad, Edward Fottrell

**Affiliations:** 1 University College London Institute for Global Health, London, UK; 2 Diabetic Association of Bangladesh, Dhaka, Bangladesh; 3 Department for Public Health Sciences, Karolinska Institutet, Stockholm, Sweden

**Keywords:** Process evaluation, Diabetes, Non-communicable diseases, Health promotion, Randomised controlled trial

## Abstract

**Introduction:**

There is an urgent need to address the growing type 2 diabetes disease burden. 20–30% of adults in rural areas of Bangladesh have intermediate hyperglycaemia and about 10% have diabetes. We report on the implementation and fidelity of a Participatory Learning and Action (PLA) intervention, evaluated through a three-arm cluster randomised controlled trial which reduced the incidence of diabetes and intermediate hyperglycaemia in rural Bangladesh. PLA interventions have been effective in addressing population level health problems in low income country contexts, and therefore we sought to use this approach to engage communities to identify and address community barriers to prevention and control of type 2 diabetes.

**Methods:**

We used a mixed methods approach collecting quantitative data through field reports and qualitative data through observations and focus group discussions. Through descriptive analysis, we considered fidelity to the participatory approach and implementation plans.

**Results:**

One hundred twenty-two groups per month were convened by 16 facilitators and supervised by two coordinators. Groups worked through a four phase PLA cycle of problem identification, planning together, implementation and evaluation to address the risk factors for diabetes – diet, physical activity, smoking and stress. Groups reported a lack of awareness about diabetes prevention and control, the prohibitive cost of care and healthy eating, and gender barriers to exercise for women. Groups set targets to encourage physical activity, kitchen-gardening, cooking with less oil, and reduced tobacco consumption. Anti-tobacco committees operated in 90 groups. One hundred twenty-two groups arranged blood glucose testing and 74 groups organized testing twice. Forty-one women’s groups established funds, and 61 communities committed not to ridicule women exercising. Experienced and committed supervisors enabled fidelity to a participatory methodology. A longer intervention period and capacity building could enable engagement with systems barriers to behaviour change.

**Conclusion:**

Our complex intervention was implemented as planned and is likely to be valid in similar contexts given the flexibility of the participatory approach to contextually specific barriers to prevention and control of type 2 diabetes. Fidelity to the participatory approach is key to implementing the intervention and effectively addressing type 2 diabetes in a low-income country.

## Introduction

Diabetes is the third leading cause of mortality worldwide [42]. An estimated 96 million people have diabetes in the South East Asian region, 90% of whom have type 2 diabetes mellitus (T2DM) [9]. 20–30% of adults in rural areas of Bangladesh have intermediate hyperglycaemia and about 10% have diabetes [32] yet awareness, treatment and control are disproportionately low [16, 27, 35]. T2DM can be prevented or delayed through a healthy diet, regular physical activity, maintaining a normal body weight and avoiding tobacco [41]. Interventions to address T2DM have tended to focus on diabetics or those at-risk [25, 36, 38], training of health workers [23] and general awareness raising [4] but these have had limited success. There is an urgent need for evidence-based population level interventions to address risk factors and acknowledge the structural and social determinants of disease [1].

We used the Medical Research Council framework to report on process evaluation (PE) [30] findings describing the implementation and fidelity of a Participatory Learning and Action (PLA) intervention evaluated through a three-arm cluster randomised controlled trial. The trial tested the effectiveness of mobile phone messaging, and PLA compared with control areas on the prevalence of intermediate hyperglycaemia and T2DM and two-year cumulative incidence of diabetes among an intermediate hyperglycaemia cohort [18]. There was a 20% absolute reduction in diabetes and intermediate hyperglycaemia prevalence and a 10% reduction in the two-year cumulative incidence of diabetes among the group with intermediate hyperglycaemia cohort in the PLA versus control arm, and the intervention was highly cost-effective [14]. Following MRC guidance, process data collection and analysis were undertaken before the trial analysis [30]. The aim of this paper is to evaluate the fidelity of the intervention to the theory and principles of the hypothesised change process [21], and examine how implementation affected the effectiveness of the intervention in order to explore the external validity of the intervention. Guidance on reporting of group-based interventions states the need for detailed reporting of implementation to understand how it affects the intervention and to enable replication [5].

### Intervention theory

The PLA intervention was inspired by the philosophy of Paulo Freire who argued that a vital precondition for positive behaviour change by marginalized social groups is the development of “critical consciousness” [20]. Critical consciousness is a process of applying critical thinking skills, as individuals examine their situation and develop a deeper understanding about their reality. Developing this understanding enables individuals to come together in the development of personal and shared confidence in their ability to improve their health [7]. There are three stages of critical consciousness [20]: 1) Intransitive thought - a fatalistic perspective when communities believe they cannot change their life situation; 2) Semi-transitive thought when communities are slightly empowered; 3) Critical transitivity where communities demonstrate the highest level of thought and action, they believe they can make changes, and they work collectively to achieve these changes through critical thinking. To reach the last stage, an active dialogical educational programme is necessary, which increases awareness about alternatives and possibilities, enabling participants to be actively involved in generating scenarios of alternative ways of being. The development of critical consciousness happens through group dialogue, and participatory action to challenge or resist the processes which place their health at risk [19].

Freire’s approach has been systematized in a community group-based PLA cycle of problem identification, planning together, implementation and participatory evaluation [37] which has been effective in reducing newborn and maternal mortality in low income countries [34]. Our intervention was an adaptation of this approach. This intervention was selected because it can support behaviour change among the most marginalised [24, 31], its effectiveness has been proven in this and similar settings, it is flexible to public health problem and context, and it can address structural and social determinants of population public health issues. On the basis of formative qualitative research, we were aware of behaviours, knowledge gaps and barriers to behaviour change, which helped us train facilitators and made us aware of some of the issues that could emerge and how they could be addressed. The problem identification phase focused on the risk factors for diabetes, how they were defined and experienced, and we explored the barriers to healthier eating practices, physical activity, and drivers of stress and tobacco consumption.

### The intervention

Thirty-two villages in four clusters (upazillas) in Faridpur District, central Bangladesh, were randomly allocated to receive the PLA intervention. Men and women had separate PLA groups to increase social acceptability, maximise participation, and account for gendered time-use and mobility. We recruited eight male facilitators for men’s groups, and eight female facilitators for women’s groups who had passed their higher secondary certificate level of education. Positions were locally advertised, and shortlisted candidates took a written test, an oral exam, and were finally selected by senior project staff and Community Advisory Committee (CAC) members. There was one CAC per upazilla with five to eight male and female members who provided feedback about the project. Facilitators were selected based on experience, communications skills, demonstrated motivation and familiarity with the area. None of the facilitators had previous group facilitation experience, but 14/16 had worked in communities for non-governmental organisations (NGOs) and 12 had worked as data collectors in our baseline survey [15]. Facilitators were paid 8000 BDT per month (around US$95).

Facilitators were line-managed by two coordinators. Coordinators had previously supervised PLA interventions on maternal, newborn and child health. They were both married women, with a Master’s level of education, living in Faridpur. Coordinators were line-managed by a District Coordinator (DM), who reported to a Senior Group Intervention Manager (SGIM). Both the DM and the SGIM had managed previous PLA interventions.

**Fig 1:**
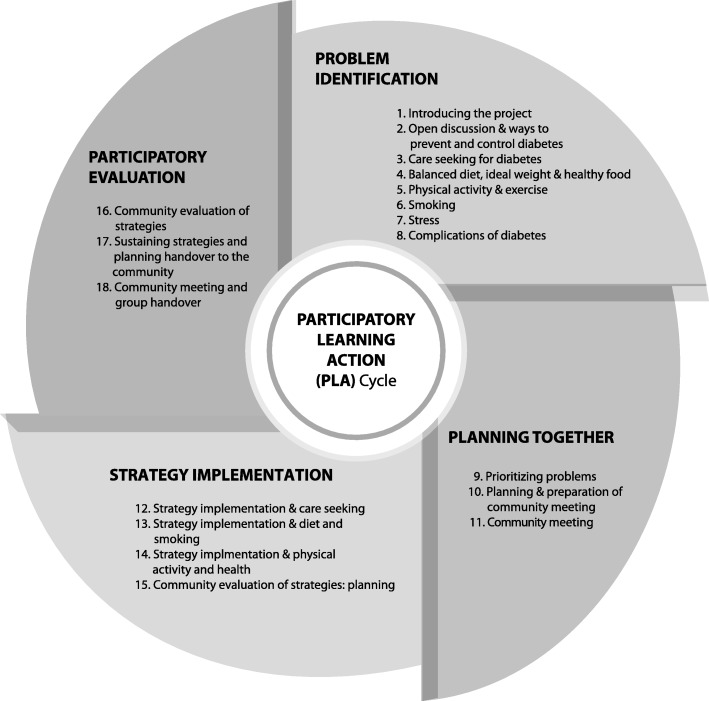
Participatory learning and action cycle

**Fig. 2:**
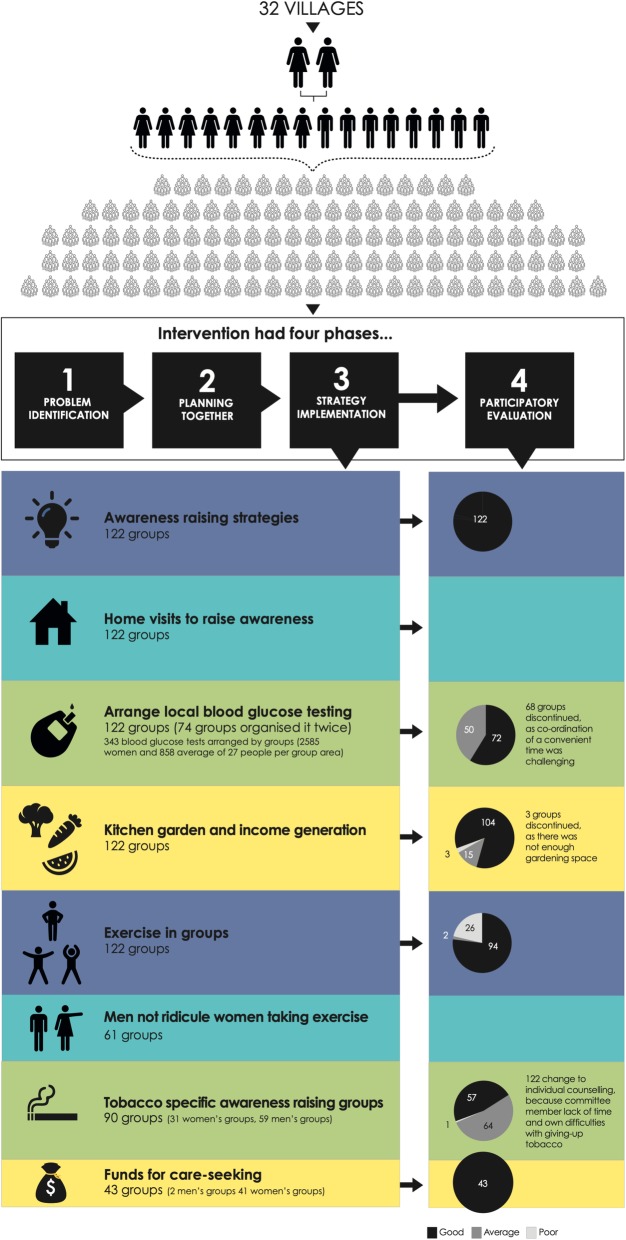
The intervention

Facilitators used a manual to guide discussions (Table 1). The intervention had four phases: problem identification, planning together, implementation and evaluation (Figs. 1 and 2). We used Diabetic Association of Bangladesh materials, and sought input on the manual design from an endocrinologist & diabetologist, a health education specialist and a nutritionist working in BIRDEM (Bangladesh Institute of Research and Rehabilitation in Diabetes Endocrine and Metabolic disorders) hospital in Dhaka. The manual was also informed by formative research [26]. For each meeting, the manual contained open questions to initiate discussions, and ‘message boxes’ of important points. Meetings had facilitation tools, such as storytelling, games or body mapping to engage participants [8] and facilitators used picture cards and a pictorial chart to explain diabetes, its causes and symptoms, and ways to prevent and control it.

**Table 1:** Meeting manual contents, methods and implementation

Phase and meeting number	Discussion content	Supplementary methods (Discussion plus…)	N groups use method	Other methods (n groups & method)
Phase 1 Problem Identification Meeting 1	Introducing the project	Game about working as a group	122	
Meeting 2	Open discussion about diabetes & ways to prevent and control diabetes	Story telling about someone with diabetes	94	28 groups: a diabetic group attender told their story
Meeting 3	Care-seeking for diabetes	Village mapping of places were care about diabetes is sought	122	122 groups: used the existing social map (made to decide the venue for group meetings) and health facilities were added to this map.
Meeting 4	Balanced diet, ideal weight and healthy food	Sort food items brought by members	34 women’s groups brought cooked foods, 88 groups brought raw fruit or vegetables	58 groups: facilitator brought oily foods to identify unhealthy foods
		Game using picture cards of food	122	12 groups: played food game using lottery as well as playing as stated in manual
Meeting 5	Physical Activity and exercise	Quiz using food plate (follow-up from meeting 4)	122	61 groups: group attenders also did the exercise
		Facilitator demonstration of exercises	122	
Meeting 6	Smoking	Game using cards	122	49 groups: facilitators told a story about a smoker
		Experience sharing (smoker/smoker in the family)	73	
Meeting 7	Stress	Story telling about someone suffering from stress	122	
Meeting 8	Complications of Diabetes	Flip chart	122	
Meeting 9	Prioritizing problems		122	
Phase 2 Planning together Meeting 10	Planning and preparation for community meeting		122	
Meeting 11	Community meeting	Drama, Story telling, Song,	122	
Phase 3 Strategy implementation Meeting 12	Strategy Implementation & care seeking		122	
Meeting 13	Strategy implementation & diet and smoking	Picture card game, nutritional plate demonstration	122	122 groups: group members brought fruit and vegetables
Meeting 14	Strategy implementation & physical activity and health	Exercise demonstration by facilitator	122	122 groups: watch a video of exercises on a laptop with demonstration from facilitator 59 groups: did the exercises
Phase 4 Participatory evaluation Meeting 15	Community evaluation of strategies: planning	Evaluation game, role play of different methods of evaluation	122	
Meeting 16	Community evaluation of strategies	Sub-committee evaluation and discussion	0	122 groups: discussion by whole group
Meeting 17	Sustaining strategies and planning handover to the community	Mapping of resources	122	122 groups: discussion about resources using map that was prepared initially 122 groups: nominated new facilitators
Meeting 18	Community meeting and group handover	Community meeting	0	122 groups: made a local committee responsible for continuing the intervention and monitoring strategy implementation, nominated facilitators were formally introduced, and given facilitation tools.

The SGIM trained the DM and coordinators on the manual content and meeting process, and they piloted meetings one to eight with four men’s groups, and four women’s groups in one non-study cluster. Piloting informed meeting length, topic sequencing and comprehension. On finalisation, facilitators were recruited and trained in phases. They received 4 days training from a diabetologist, and a nutritionist about diabetes prevention and control. The SGIM trained facilitators on PLA, community entry, and meetings one to eight (phase 1 problem identification) over 4 days. They subsequently received 4 days training for phase two (planning together) and three (implementation), and 2 days training for phase four (evaluation). Coordinators each supervised eight facilitators through monthly meetings in Faridpur, and community observation. Facilitators also used their own tools and methods and shared ideas in monthly meetings.

We planned a minimum coverage of one group per 200 population aged ≥ 30 years with at least one men’s and one women’s group in each intervention village. The requirement to have separate men’s and women’s groups resulted in a higher population coverage than planned, with 1 group per 145 population aged ≥ 30 years (range: 101–199). We engaged with village leaders and community members in each village to make social maps of household clusters, mosques and market areas to identify the most appropriate venues for group meetings. Coordinators and facilitators visited households to spread information about the groups and organized meetings in venues and at times convenient to participants. There were 122 groups facilitated by 16 facilitators, and each facilitator was responsible for 6 to 9 groups each month. Group attenders were not given any incentives.

## Methods

### Setting

Faridpur is around 2000 km^2^ with a population of over 1.7 million, and a mainly agricultural economy of jute and rice farming. Primary healthcare is provided at the village level through Community Clinics (CC) and Family Welfare Centres (FWCs) [29] who have received diabetes screening and referral training. Glucometers and blood glucose testing strips should be available at CCs and FWCs but re-supply is irregular, and blood glucose testing was not routinely available. Village level private health care is available through informal health workers and drug vendors who provide blood glucose tests. Services for diabetics are provided in upazilla health complexes, and in Faridpur headquarters at the Diabetic Association of Bangladesh hospital, but these are too far away for many diabetics. There were 14 CCs, 22 FWCs, and three upazilla health complexes in PLA intervention areas. The population in Faridpur is mainly Bengali and 90% are Muslim [3]. 8.9% of men and 11.4% of women aged ≥30 years have diabetes with only 24.6% being aware of their status, and 75% of known diabetics had sub-optimal control [16].

### Data collection

The intervention was participatory and complex and therefore we used the Medical Research Council framework [21] for process evaluation research to 1) evaluate the fidelity of the intervention to the participatory theory and method 2) describe the implementation of the intervention, and 3) explore how the implementation of the intervention affected its effectiveness. We used structured observation, narrative observation, and focus group discussions to collect data using a concurrent nested mixed-methods research design [11]. We collected qualitative and quantitative data at the same time and used qualitative data to validate and explore quantitative results every 4 months. Facilitators recorded attendance on paper forms and presented reports to coordinators. Coordinators supported facilitators, and planned to observe and collect data at a minimum of 30 meetings per month. Coordinators conducted narrative and structured observation of facilitators at these meetings to explore fidelity to the participatory method. Coordinators used structured observation to give facilitators scores out of 10 on how questions were posed; use of participatory tools; and how successful facilitators were in holding group attention and participant interest. Holding group attention was evaluated by observing side-talk, attention and participation in discussions. Every form had open questions about group plans, challenges (such as village rivalry, farming activities), opportunities (an upcoming community event, or implementation of a new method) and the discussion agenda. Coordinators attended every community meeting in the planning together phase and recorded prioritised problems and planned strategies on paper forms. In the implementation and evaluation phases facilitators used paper forms to record the strategies implemented, strategies evaluated, and the results of the evaluation. There was some responsive design of quantitative paper forms to quantitatively capture what was happening in the groups (for example to record how many group members had spoken about the meeting agenda with others), but the dynamic group participatory process didn’t lend itself easily to systematic quantitative data capture.

From previous PLA trials, we hypothesized that the retention of facilitators and supervisors, and the frequency of supervision may affect the implementation of the intervention. This data was collected by the PE manager and documented in quarterly reports.

The PE manager (KAk) observed 2–6 meetings, four to six times a year and interacted with attenders, non-attenders, facilitators and coordinators, taking detailed field notes. Field observations and qualitative data collection were guided by four research questions: What affects group attendance? What are the differences and similarities between men’s and women’s groups? What do the groups find challenging and enjoyable and why? To what extent do group attenders, facilitators and coordinators feel the intervention is being effective and why? KAk conducted a focus group discussion (FGD) with two coordinators at the beginning of phase three, and two FGDs with facilitators at intervention completion. She used a topic guide based on the four research questions detailed above.

### Data management and analysis

Paper forms were collected and checked monthly by the DM and by KAk. Data inconsistencies were reconciled by phone. Quantitative data were entered into Excel and summarized in quarterly reports alongside data from open questions and field observations. JM and KAk discussed and analysed reports, discussed further research questions to be explored and iteratively planned the next phase of data collection. KAk conducted and recorded FGDs in Bangla. She wrote a narrative report in English with descriptive quotes of the coordinators’ FGDs. KAk transcribed and translated facilitators’ FGDs into English. FGD data were analysed by hand according to emergent codes and codes focused on the four research questions (stated above) by KAk and JM. HJ analysed FGDs independently and findings were compared and discussed with JM and KAk. JM and KAk wrote a results narrative of findings, which was read by HJ, EF and CK to check for consistency.

## Results

### Location and attendance

**Fig. 3:**
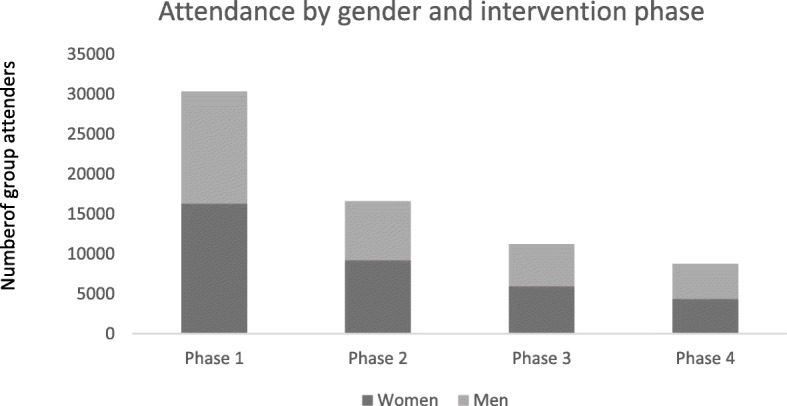
Attendance by gender and intervention phase

The PLA intervention was implemented from July 2016 to December 2017. Sixty-one men’s group and 61 women’s group meetings were conducted every month. Attendance was highest in phase one, with an average of 39 women, and 33 men per group in meetings one and two. Attendance stabilised over subsequent phases with an average of 24 attenders in phase four, with slightly higher attendance of women than men (Fig. 3).

### Facilitator retention

Six facilitators (3 female and 3 male facilitators) resigned during the intervention, leaving after the 3rd (*n* = 1), 9th(*n* = 1), 12th (*n* = 1) and 16th (*n* = 3) month. Three facilitators (one female and two male) resigned because of alternative employment opportunities. One woman resigned because of pregnancy and another left because her husband forbade her to work outside of the home. Three replacements were selected from a reserve list, one of which was a group attender. Other replacements were group attenders. Replacements worked with the outgoing facilitator for 1 month and received on-the-job-training.

### Implementation

#### Phase 1 introduction to diabetes and barriers to behaviour change (problem identification)

Groups discussed diabetes and diabetic care, the barriers to preventing and controlling diabetes and potential strategies to overcome barriers over 8 months. They planned a community meeting to interact with non-attenders, village leaders and health workers and get support to implement chosen strategies. Each coordinator observed an average of 21 meetings per month in the first 2 months, and thereafter this increased to 33 meetings per month. Coordinators tended to observe meetings where the facilitator was less skilled, or they needed support with a context-specific issue.

**Fig. 4:**
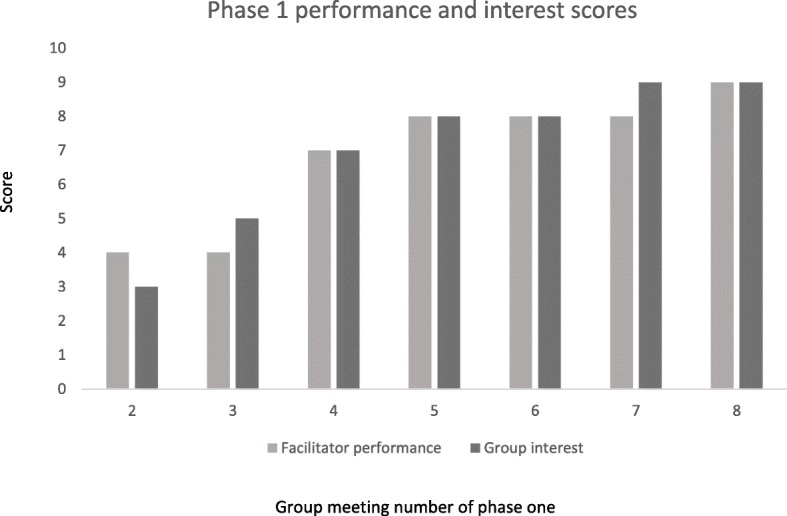
Facilitator performance and group attender interest

Figure 4 shows that group interest increased from meeting two to meeting eight in phase one, and facilitators were more able to conduct meetings in a fully participatory way as time progressed. Attenders requested refreshments and blood glucose testing at first, but these demands were infrequent after meeting four: “At the beginning (encouraging attendance) was a problem. Locals wanted us to arrange free blood glucose tests. But we convinced them and later, they understood (the benefits of attending the meeting).” (Coordinator Boalmari and Madhukali).

**Fig. 5:**
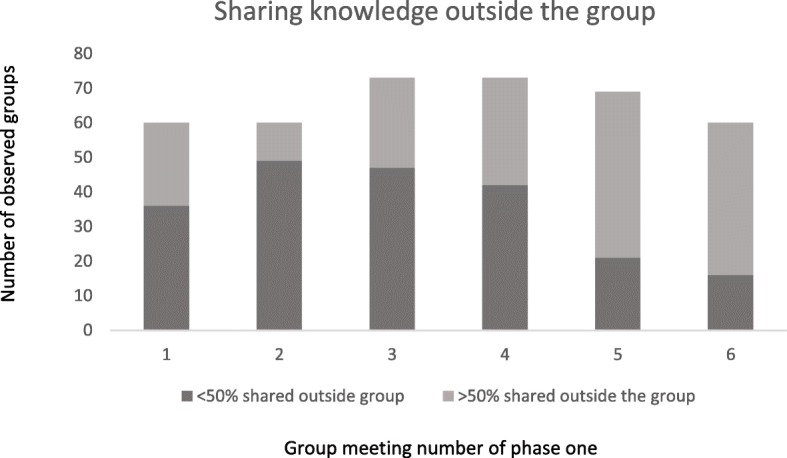
Sharing knowledge outside the group

Facilitators encouraged attenders to discuss meeting topics within their households and the wider community. Group attenders described their own behaviour change and sharing of meeting topics through a show of hands. By meeting seven, > 50% of group attenders in 80% of observed groups had shared discussions with the wider community (Fig. 5). By the end of phase one, > 50% of group attenders in 53% of groups had either started exercising, changed their eating habits or started kitchen gardening. A coordinator explained: “I have seen that a few local people of that village have brought a gourd plant to grow in their garden. They plan to eat and sell them.” (Coordinator Boalmari & Madhukhali).

#### Phase 2 planning together

This phase took 4 months. Facilitators suggested that groups from one village jointly plan and conduct a community meeting, presenting prioritised problems and discussing community strategies to address them. Between two and six groups from each village conducted 32 joint community meetings. When a few Muslim and Hindu women’s groups were reluctant to meet together, facilitators and coordinators worked with these groups to find acceptable venues. Each group nominated a committee of three to ten attenders who met between meetings 9 and 10 to plan the community meeting. Committees from 15 groups did not have a planning meeting and the facilitator coordinated planning. Facilitators ensured that both men and women participated in planning the venue, discussion topics and meeting format. Village leaders (local politicians, imams, teachers, retired government officials), health workers, and government officers were invited to community meetings by facilitators or coordinators.

Community meetings took place between 4th April and 21st May 2017, in the afternoon. All 32 villages held meetings with an average attendance of 316 participants (total attendance = 10,120). All three invited government officials attended community meetings, and 38/90 invited government health workers, 31/59 NGO workers, and 110/180 local leaders attended meetings. 40% (4017/10120) of those attending were group attenders, and more women attended than men (57% vs. 43% respectively).

Community meetings took place in schools, madrasas (Islamic educational institutes) or the courtyard of a house. Coordinators attended all meetings, and the SGIM observed five community meetings. Each group was given 1500 BDT (18 USD) to rent chairs, a tent, and a microphone, or for participant refreshments, government officials’ allowances (according to locally agreed and expected rates) and payment to those group members who spent substantial time doing local coordination and rental of equipment.

At the community meeting, attenders introduced the group intervention, discussed diabetes symptoms and risk factors, and presented information about care-seeking for diabetes. All groups used the chart and picture cards, and a drama to present risk factors and social barriers to healthy behaviours. When community leaders participated in the drama this was particularly well received: “One Imam agreed to play a role himself in the drama which was really surprising.” (Coordinator Boalmari and Madhukhali). Coordinators and some group members sang diabetes songs they had composed in the community meeting. Groups presented the prioritised barriers to healthy behaviours, and suggested strategies to overcome these problems. Strategies were discussed and communities drafted action plans.

Observation data show that discussion and questions about diabetes and risk factors was common, and that communities were keen to discuss and carry out action plans. Community meetings generally had a positive impact on the group and the village response to the group: “Conducting the community meeting was really helpful. Large numbers of people came and so they knew about our activities, and some were inspired to attend the next meeting.” (Coordinator Boalmari and Madhukhali). Coordinators reported that where household decision makers attended the community meeting, it was easier for women in particular to come to subsequent meetings. Coordinators also noted that the community meeting helped build relationships with non-attenders, and attendance of a village leader endorsed the work of the group making strategy implementation easier: “If a community leader tells people to attend the meeting then they listen to them and attend” (Coordinator Nagarkhanda and Saltha). There were only a few challenges in conducting community meetings. Village conflict affected a few meetings, and some men didn’t attend because they felt disrespected by being asked to sit on the ground. Women’s participation was more difficult when the meeting was near a mosque or far from their home, and working men found it difficult to attend.

#### Phase 3 implementation

For 4 months after the community meeting, groups implemented strategies and continued discussing diabetes risk factors, prevention and control. While many resource persons were named at the community meeting, in practice, group attenders took responsibility for strategies. Table 2 shows the problems and strategies that were prioritised in community meetings and implemented by groups. Facilitators felt that the strategies were important to the success of the intervention: “Strategies are needed for everything. Strategies help to communicate with people. Without strategies it is difficult to get a good result” (Facilitator FGD).

**Table 2:** Prioritised problems and planned and implemented strategies

Problem	Strategy	N groups	Problem category
Lack of knowledge about glucose testing to prevent and control diabetes	Awareness raising through household visits by small groups of group attenders	122	Diabetes knowledge and care
Glucometer and/or glucometer testing strips are unavailable in villages, and it is unaffordable to travel to Faridpur head quarters.	Use the group fund to travel to test blood glucose levels or to visit a medical professional.	43	Diabetes knowledge and care
Local blood glucose testing is not available	Facilitator to arrange village measurement of blood glucose	122	Diabetes knowledge and care
Lack of knowledge about the need for a balanced diet (in quantity and type of food)	Awareness raising through household visits by small groups of group attenders	122	Diet
Vegetables are not always available, and it is not always feasible to buy in large quantities	Kitchen gardening and income generation (such as selling vegetables, and rearing livestock) to increase household access to vegetables	122	Diet
Lack of knowledge about relation between physical activity and diabetes, and the sufficiency of exercise to remain healthy	Awareness raising through household visits by small groups of group attenders, including counselling of home-based physical exercise for women.	122	Physical Activity
Cultural taboo towards women walking outside their home Lack of knowledge about swimming as exercise Lack of interest and motivation	Exercise in groups, led by group attenders	122	Physical Activity
Men ridicule women walking outside their homes	Men commit to not ridicule women walking outside their homes	61	Physical activity
Lack of knowledge about the effect of smoking among men and its relationship with diabetes	Awareness raising by small groups of group attenders	61	Smoking and tobacco
Men and women are addicted to tobacco products, so it is difficult to give up	Group attenders, and those who have given up tobacco products personally encourage tobacco users to give up.	122	Smoking and tobacco
	Support for men and women to stop consuming tobacco products through anti-tobacco sub-committees making household visits	90	Smoking and tobacco
Smoking is promoted by peer pressure	Male group attenders will encourage adolescents not to smoke	61	Smoking and tobacco

### Awareness raising

#### Anti-tobacco committees

The most common barrier to healthy lifestyles was lack of knowledge, and therefore all groups sought to increase awareness through forming smaller groups to conduct household visits. Anti-tobacco committees were formed in 90 groups (31 women’s and 59 men’s groups), with women focusing on smokeless tobacco consumption, and men focusing on cigarettes and smokeless tobacco. These committees were to visit households, raise awareness and support users to quit.

#### Engaging households about exercise and diet

General diabetes awareness raising also occurred through household visits by small groups of attenders who discussed diabetes risk factors and how to have a healthy lifestyle. Attenders volunteered for these visits at meetings, and groups set targets for the number of people encouraged to start physical activity, kitchen-gardening, cooking with less oil, or stopping smoking and tobacco consumption. Attenders reported on these targets at each meeting. Men’s and women’s groups planned and implemented home-visits separately, with women’s group attenders discussing healthy lifestyles with the women of the household, and men’s group attenders discussing with men. Observation data indicated that when men and women from the same household attended meetings, dietary behaviour change was easier because of the gendered nature of household activities. For example, men usually bought the vegetables and women usually prepared and cooked them. If both attended groups, they could manage household behaviour change together, using less oil and salt and eating more vegetables: “Women attenders said that if curry does not taste good because of less oil then their husbands scold them…but men are accusing women of using too much oil.” (Coordinator Nagarkhanda and Saltha).

Facilitators also participated in awareness raising strategies. All facilitators (male and female) approached Imams and asked them to discuss healthy behaviours, prevention and management of diabetes in their Friday sermons: “In two of my villages there are imams who discuss the meeting and its content in the mosque on Friday. People are now more conscious.” (Facilitator FGD). Twelve imams discussed diabetes prevention and control in the mosque. Imams came to mixed religion groups, and Muslim only groups, and discussed religious aspects of healthy behaviours. In mixed groups there was also some discussion about Hindu practices that helped prevent and control diabetes.

#### Physical activity

Women’s and men’s groups formed physical activity sub-groups to encourage exercising together, and address gender barriers preventing women from walking: “If anyone faces family problems in going for a walk, another attender does it with them. This is how the team works.” (Facilitator FGD). Men and women rarely walked together, but men discussed their commitment to supporting women exercising, and many women who had previously not undertaken any conscious physical activity began exercising: “Five attenders were overweight in my group. All of them were had intermediate hyperglycaemia. One started to walk, and she improved. Others saw what happened to her, and they started to walk and maintain their diet and they also got a good result.” (Facilitator FGD).

#### Group funds and income generation

Funds were initiated by 2 men’s groups and 41 women’s groups. Thirty-one groups had initiated funds before the community meeting, 3 to 4 months into the intervention. Coordinators reported: “Most men are not interested in making a fund. They have access to money and if they need money they borrow from their neighbors, so they do not need a fund. Another reason is they found no one trustworthy to manage a fund.” (Coordinator Nagarkhanda and Saltha). Coordinators had received fund management training in previous interventions, and they trained facilitators who then helped groups. Groups decided on fund deposit mechanisms and amounts and not all attenders deposited money. On average, 13 group members per group contributed to the fund, paying 20–50 BDT (0.24–0.6 USD) per month. Groups appointed a cashier and assistant cashier who kept the fund, and maintained a register of deposited, lent and repaid money. In all groups, at least four attenders had to consent before money was lent. New attenders were expected to put in the equivalent money that had been deposited already by other attenders before they could borrow money. By the end of phase four the funds had an average of 2346 BDT (28 USD) per group. Money from the fund was usually lent for a short period of time to buy food, plants or seeds, medicine, for maternal and child health, travel for blood glucose testing or funding the test itself.

One of the perceived barriers to eating a healthy and balanced diet was cost. Many groups promoted eating eggs as a meat alternative, and kitchen gardening and income generation activities (such as selling vegetables, and rearing livestock) increased household access to vegetables. Women usually planted and tended kitchen gardens and cared for livestock, and men usually bought the seeds and bought and sold the livestock.

#### Blood glucose testing

At every community meeting, people wanted to make blood glucose testing available in villages. 74/122 groups arranged blood glucose testing twice during phase three. By meeting 18, 3343 blood glucose tests had been arranged by groups (2585 women and 858 men, mean = 27 people per group area). More women than men were tested because men tended to be working when testing occurred. Facilitators usually contacted an informal health provider to bring testing strips and a glucometer to a local place at an arranged time. Informal health providers referred those with intermediate hyperglycaemia or diabetes to the diabetic hospital in Faridpur and advised them to follow lifestyle advice given in group meetings. We do not have data on how many people went to the diabetic hospital after this test. Some people who went to Faridpur found that their blood glucose level was normal and were annoyed: “Those people reacted to this and accused the informal health worker of harassing them.” (PE Observation report, June 2017).

Groups were unable to make changes in the government health care provision for diabetes at the local level, despite enthusiasm for this at community and group meetings. A local politician who attended group meetings, and an upazilla health and family planning officer were unsuccessful in attempts to make blood glucose testing available at local CCs after the community meeting. Re-supply of testing strips was not addressed and facilitators also heard about community mistrust of health workers: “When villagers go to the CC, (health workers) behave badly towards them and so people don’t want to go there. I think the staff are less experienced and that’s why they do this.” (Facilitator FGD). People from 20 villages served by five CCs sought blood glucose testing services at CCs after the community meeting, but only received services at one CC.

### Phase 4 participatory evaluation

Groups reflected on their progress in addressing barriers to healthy behaviour over 3 months. 38/122 groups evaluated strategies with non-attenders, and 84 groups evaluated strategies through self-reflection. Four groups with targets for strategies evaluated according to these targets. For example, if only 10 people out of a targeted 20 had started exercising regularly, this was considered average performance (Table 3). Other groups evaluated strategies by evaluating against their own definition of success. With some strategies, groups did not reflect on whether their strategy had affected the identified barrier. For example, the fund was not used frequently, but groups evaluated it against criteria of its continued existence, and regular contribution of attenders. Harassment was a barrier to physical activity for women, but groups did not evaluate the extent to which harassment had decreased. Those groups who engaged with a health worker or a politician did not evaluate these strategies. After evaluation, all groups decided to continue awareness raising, and group physical activity. All groups with a fund (*n* = 43) decided to continue this strategy. No groups added strategies. Coordinators felt that groups needed more time to implement their strategies fully before evaluating them: “Two years is too short for group activities.” (PE Observation notes, October 2017).

**Table 3:** Evaluated strategies

Strategy	Performance			N groups evaluated	N groups continued	Reason
	Good	Average	Poor			
Awareness raising	122			122	122	N/A
Physical activity	94	2	26	122	122	N/A
Anti-tobacco committees	57	64	1	122	122 Changed to individual counselling	Committee member lack of time and own difficulties with giving-up tobacco
Income generation & kitchen gardening	104	15	3	122	119	Not enough gardening space
Blood glucose testing	72		50	122	54	Co-ordination of a convenient time was challenging
Fund	43			43	43	N/A

When funded support for the group meeting came to an end, a handover community meeting was proposed but attenders were too busy with farm work. Instead, groups invited two or three village leaders to attend a handover meeting and request support in future planning. Groups nominated a volunteer facilitator, and they received facilitation training. The volunteer facilitator was confirmed at the handover meeting, and most groups said they would continue meeting.

## Discussion

Intervention implementation is integral to its’ success or failure [12] and comprehensive reporting can enable interventions to be transferred to different settings [5, 22]. We evaluate the fidelity of the intervention to the theory-driven method, explore how implementation affected the effectiveness of the intervention and discuss how this affects the external validity of the intervention.

### Fidelity to participatory methods

We expected high fidelity to participatory methods within groups because senior staff and coordinators were experienced in the methods, tools and approach, and, based on our experience with PLA interventions, attenders feel more comfortable participating over time, as they become familiar with each other and the method. An experienced senior team led to strong and consistent mentoring and motivation of facilitators, and meetings were conducted in a progressively participatory way. In order to develop the skills to supervise participatory approaches, more time should be spent in the formative project phase developing communication skills and being mentored in the development of participatory skills. Previous group-based interventions have had lower attendance than reported here [17]. High attendance could have hampered participation in methods and games, but we did not observe this.

### Fidelity to the method of raising critical consciousness

Formative research and the process of problem identification enabled critical reflection about the determinants of behaviours among attenders, facilitators, coordinators and the senior team. All groups received active dialogical education throughout the intervention, conducted the planned number of meetings, took action, and reflected on their progress. Groups implemented similar strategies because (1) Our formative research showed that the barriers to healthy behaviours were similar across study areas and (2) Groups were keen to act, but often unsure about what to do. Strategy examples were given in the manual, and between-group sharing of ideas was enabled by coordinator and facilitator meetings. In order to engage with community and systems barriers more effectively, future interventions implemented over a longer time could include examples of policy and advocacy approaches to address issues such as blood glucose testing at CCs. This could include tools and methods such as photovoice, film and/or theatre to communicate with policy makers and advocate for change [28, 33, 39]; mapping of policy stakeholders and local champions to advocate for systems change [6]; providing information to coordinators about national policies and plans to enable community and policy efforts to act in synergy [40]; and specific capacity building to enable coordinators and SGIM to support group engagement with policy makers and health workers.

### External validity of the PLA approach

Population-based approaches to the prevention and control of T2DM have been found to have equal benefits to medication-based approaches, and wider benefits in preventing diabetes among those not yet at risk [2]. Exercise and diet interventions have shown some success in preventing and controlling T2DM, with better effects where both behaviours were targeted, and delivered in a group environment [10]. There is also some evidence for the effectiveness of peer-support interventions [13], but more research is needed in low-income countries as a recent cluster randomised controlled trial of a peer-support intervention targeting high risk groups in Kerala did not reduce diabetes incidence after 24 months [38]. There is also limited evidence of population level interventions in low-income countries. Our intervention took a population level, socio-ecological approach, addressing the social and contextual barriers to healthy behaviours. In order to support socio-contextual and norm changes, action by a variety of community, household and individuals is necessary, not just those at risk. The intervention is complex, but it is cost effective [14] and, given that it is focused on changing the social context, may have sustained health effects. The WHO acknowledges the need to engage communities in the prevention and control of non-communicable diseases, and this intervention offers one way to do this effectively [43].

While many interventions conduct formative research to inform the development of interventions, the participatory approach ensures that contextual adaptation can occur throughout the intervention and it retains relevance to attenders and community members. The interaction of the intervention with actors and contexts is, in fact, a key component of the intervention. When considering the external validity of the intervention, fidelity to the ‘function’ – the participatory approach – as opposed to the ‘form’ of the intervention will be important, enabling time for attenders and non-attenders to develop knowledge, realisation of their situation and become motivated to take action [21]. Precisely because the intervention is participatory means that it may have similarly positive results in other contexts, using different community based strategies to address different community based issues, but through a participatory approach. Future research could replicate the PLA intervention in different contexts to understand its effectiveness, as some contexts might be more amenable to PLA than others. Evidence from the maternal and newborn health literature [34] suggests the adaptability of the PLA approach to context and topic. Another area of research priority is exploring optimal governance arrangements for training, supervision and implementing the intervention at scale in Bangladesh, as well as building contexts and capacities for further engagement with health systems.

### Limitations

We sought to collect longitudinal data from as many groups as possible, without adding burden to facilitators and coordinators which limited our methods. Data collection methods to evaluate the extent of participation were somewhat crude and subject to social acceptability bias with coordinators potentially motivated to report positively as this also reflected on their performance. Triangulation of quantitative findings through observation data and FGD data added rigor.

## Conclusion

Our supervisory approach combined with experienced and committed senior staff were key in maintaining fidelity to a participatory methodology, and additional time, capacity building and support could enable engagement with systems barriers to behaviour change. Our intervention was implemented as planned and was largely aligned with the theory-base of the intervention. We support the development of replication trials in other contexts to test the effectiveness of the PLA approach to address the increasing global burden of type 2 diabetes in low-income countries.

## Data Availability

A data sharing committee will review requests for data on a case by case basis.

## Abbreviations

BIRDEM: Bangladesh Institute of Research and Rehabilitation in Diabetes Endocrine and Metabolic disorders
CAC: Community Advisory Committee
CC: Community clinic
DM: District manager
FGD: Focus group discussion
FWC: Family Welfare Centre
NGO: Non governmental organisation
PE: Process evaluation
PLA: Participatory learning and action
SGIM: Senior Group Intervention Manager
T2DM: Type 2 diabetes mellitus
